# Evaluating the safety and efficacy of rapid bolus-only dexmedetomidine for pediatric EEG sedation: a retrospective study

**DOI:** 10.1007/s00431-025-06567-x

**Published:** 2025-11-18

**Authors:** Yael Biro, Yaacov Tabi, Alex Gileles-Hillel, David Rekhtman

**Affiliations:** 1 Pediatric Emergency Department, Mt. Scopus Campus, Hadassah Medical Center, Mt. Scopus Campus, Jerusalem, Israel; 2 Pediatric Department, Mt. Scopus Campus, Hadassah Medical Center, Jerusalem, Israel; 3 Pediatric Pulmonology and Sleep Unit, Hadassah Medical Center, Jerusalem, Israel; 4 Faculty of Medicine, Hebrew University of Jerusalem, Jerusalem, Israel; 5 Pediatric Sedation Service, Mt. Scopus Campus, Hadassah Medical Center, Jerusalem, Israel

**Keywords:** Dexmedetomidine, Pediatric sedation, EEG, Bolus administration

## Abstract

Dexmedetomidine is an α2-adrenergic receptor agonist known for its sedative and analgesic properties. It produces an EEG pattern resembling Stage II sleep without interfering with EEG interpretation, making it a preferred agent for pediatric sedation, particularly during EEG procedures. Traditionally administered as a bolus followed by continuous infusion, this approach can challenge workflow efficiency and staffing in resource-limited settings. This study aimed to evaluate the safety and efficacy of a rapid bolus-only dexmedetomidine protocol for pediatric EEG sedation. This retrospective study included pediatric patients aged 1–18 years undergoing EEG with sedation at the Hadassah Medical Center between 2015 and 2023. All patients received dexmedetomidine via intravenous bolus (1–2 mcg/kg), with additional doses (0.5–2 mcg/kg) as needed. No continuous infusion was used. Primary outcomes were sedation success rate, time to onset and recovery, and adverse events. A total of 345 patients were included. Sedation was successfully achieved in 99% of cases, with a median sedation onset of 5 min. The overall adverse event rate was 8.4%, with no sentinel events. Hypotension and hypoxia occurred in 1.7% of patients each. Patients with upper airway abnormalities were more likely to require additional doses (*P* = 0.001) and showed a trend toward increased adverse events (*P* = 0.095). Adverse event risk increased with higher total dexmedetomidine doses (≥ 3 mcg/kg, *P* = 0.001).

*Conclusion*: Rapid bolus administration of dexmedetomidine without continuous infusion provides effective sedation for pediatric EEG, with a good safety profile, similar to other sedation protocols. This cost-effective approach offers significant advantages for settings with limited staffing or high patient turnover.

**Table:** 

**What is Known:** • *Dexmedetomidine is well known as an effective agent for EEG procedural sedation*.
**What is New:** • *We present a novel IV single bolus protocol utilizing dexmedetomidine that demonstrates a high success rate in achieving adequate sedation, is associated with a low incidence of adverse events, and requires an overall lower dosage compared to traditional methods*.

**What is Known:**

• *Dexmedetomidine is well known as an effective agent for EEG procedural sedation*.

**What is New:**

• *We present a novel IV single bolus protocol utilizing dexmedetomidine that demonstrates a high success rate in achieving adequate sedation, is associated with a low incidence of adverse events, and requires an overall lower dosage compared to traditional methods*.

## Introduction

Electroencephalography (EEG) is a common exam in pediatrics, requiring the child to remain still throughout the procedure. The need to capture EEG during spontaneous sleep further complicates the process. Children with developmental delays or behavioral disorders often require procedural sedation to obtain a quality EEG [1].

Commonly utilized sedative agents for pediatric neurodiagnostic investigations include chloral hydrate (CH) and dexmedetomidine (DEX). CH has a longstanding history of use in pediatric procedural sedation, characterized by a favorable safety profile [2] and minimal impact on EEG waveform [3]. However, its clinical utility is limited by a prolonged onset time to achieve adequate sedation (30–60 min) [4], and a higher failure rate in children older than four years [5] and those with neurological disorders [2]. DEX is a selective alfa-2 adrenergic agonist with a short half-life. Its effects on the central nervous system include sedation and anxiolysis, and a sympatholytic effect leads to decreased heart rate and blood pressure [6]. DEX sedation elicits an EEG pattern similar to that of Stage II sleep, and does not hinder EEG interpretation [7], making it a useful agent for EEG sedation in children. DEX can be administered via intravenous (IV), intramuscular (IM), or intranasal (IN) routes, each with its advantages and disadvantages. IN and IM routes offer the benefit of easier administration, as they do not require IV access; however, they are associated with a prolonged and less predictable onset of action, as well as a higher sedation failure rate compared to IV administration [8]. In time-sensitive settings, the delayed onset of action with IN or IM routes is particularly problematic if the initial DEX dose is insufficient to achieve full sedation, necessitating additional waiting time.

Traditionally, intravenous DEX is administered using a loading dose of 0.5–2 mcg/kg delivered over 5–10 min, followed by a continuous infusion at 0.5–1 mcg/kg/h [6]. However, due to limited medical staff availability, a need arose to develop a sedation protocol that would support efficient patient turnover while maintaining safety and effectiveness. To address this, we implemented a protocol involving rapid bolus administration of DEX without a subsequent continuous infusion.

The objective of this study is to evaluate the safety and efficacy of this bolus-only DEX sedation protocol for pediatric patients undergoing EEG. To our knowledge, this is the first evaluation of this specific administration strategy. By assessing sedation success rates, recovery times, and adverse events, this study aims to provide evidence supporting the feasibility of this simplified sedation approach in clinical practice.

## Methods

This single-center, retrospective study was conducted at the Pediatric Department of Hadassah University Hospital—Mount Scopus, Jerusalem, Israel, from September 2015 to May 2023. The study included all patients aged 1 to 18 years who were referred for EEG under sedation due to behavioral disorders or failure to obtain an adequate EEG recording during sleep deprivation. The Pediatric Neurology Unit at Hadassah Mount Scopus performs approximately 1,000 EEG examinations annually, with around 100 conducted under sedation. Notably, most of the children requiring sedation have an underlying diagnosis of global developmental delay (GDD) or autism spectrum disorder (ASD).

This study was conducted in accordance with the Declaration of Helsinki and was approved by the institutional ethics committee of Hadassah Medical Center (HMO-0442–23). The requirement for informed consent was waived due to the retrospective nature of the study.

### Data collection and patient evaluation

Data were collected from medical records, including: Patient demographics (age, sex, chronic medical conditions, pre-sedation evaluation, and chronic use of prescription drugs), and sedation-related parameters (number of intravenous DEX doses administered, total DEX dose per kilogram of body weight, time to sedation onset, time to sedation offset, and adverse event profile).

For analysis purposes, all patients with any upper airway condition—whether acute or chronic (e.g., stridor, obstructive sleep apnea (OSA), tracheal stenosis, tracheostomy, snoring, or rhinorrhea)—were categorized under the group of upper airway abnormalities.

All patients underwent a pre-procedural evaluation by a pediatrician. Sedation procedures were performed by the head of the pediatric sedation service, who is a pediatric emergency medicine specialist. The exams were performed in a designated sedation room equipped with continuous monitoring, including: Pulse oximetry, electrocardiography (ECG), and non-invasive blood pressure measurement, wall-mounted oxygen supply, airway management devices, suction apparatus, defibrillator, and resuscitation medications.

### Sedation protocol

Sedation team consisted of sedation provider, sedation nurse and EEG technician. All dedicated and experienced team members.

In order to minimize patient discomfort and anxiety, the procedure was held in a dedicated EEG suite, with dimmed light and a quiet atmosphere. Parental presence was encouraged until the child was fully sedated. Whenever feasible, topical anesthesia was applied prior to venipuncture. No restraints were used.

After IV placement, sedation was administered, and continuous monitoring for any adverse events continued throughout the exam and until the child was awake and back to their basic status. EEG recordings were performed by an EEG technician, while continuous vital sign monitoring and adverse event documentation were conducted by a sedation nurse.

Sedation was initiated with a rapid intravenous bolus of DEX (1–2 mcg/kg) administered over 1–2 min, followed by additional boluses (0.5–2 mcg/kg) as needed to achieve the target sedation level. The target sedation level was defined as a Motor Activity Assessment Scale (MAAS) score of 3 (calm and cooperative).

If target sedation was not achieved after three doses of DEX, a rescue dose of intravenous promethazine was administered before determining sedation failure. Continuous DEX administration was never part of the protocol.

Agitation during EEG recording was an indication for additional bolus administration. The duration of the EEG ranged from 20 to 40 min. Post-procedure, parents were promptly invited back into the room to facilitate the recovery and awakening processes in a secure and familiar environment. Recovery was conducted in the same setting as the EEG to ensure continuity of care.

### Study outcomes

The primary outcomes of the study were the efficacy and safety of rapid bolus DEX administration. Efficacy was assessed based on the time to achieve adequate sedation, the number of failed sedation attempts, and the time to complete recovery and discharge. Safety was assessed based on the documentation of adverse events, classified as sentinel, moderate, minor, or minimal according to the World Society of Intravenous Anaesthesia (SIVA) Adverse Sedation Event Reporting Tool. Per SIVA criteria, alterations in vital signs—including bradycardia, tachycardia, hypotension, and hypertension—are defined as changes exceeding 25% from baseline. However, because the majority of patients in this cohort had autism spectrum disorder (ASD) accompanied by behavioral challenges and limited cooperation, it was often not feasible to obtain reliable baseline resting vital signs. Therefore, the following adapted definitions were used: blood pressure alteration was defined as systolic blood pressure below the 5th percentile for age. Heart rate and respiratory rate alterations were defined as deviations from the age-specific normal ranges established by the American Heart Association Pediatric Advanced Life Support (PALS) guidelines. Hypoxia was defined as an oxygen saturation between 75–90%. Severe hypoxia (< 75%) was defined as a sentinel event.

### Statistical analysis

Categorical variables were compared using the chi-square (χ^2^) test or Fisher’s exact test. Continuous variables were analyzed using the Student’s t-test for normally distributed data and the Wilcoxon-Mann–Whitney test for non-normally distributed data. A two-tailed P-value of < 0.05 was considered statistically significant. Statistical analyses were performed using SPSS version 29.0.

## Results

A total of 345 patients (242 males, 103 females) were included in the study. The median age was 5 years (range: 1–18 years; interquartile range [IQR]: 5 years). Among the cohort, 274 patients (79%) had a diagnosis of global developmental delay (GDD), 211 (61%) had autism spectrum disorder (ASD), and 174 (50.4%) had epilepsy.

Pre-sedation risk factors included congenital heart defects in 10 patients (2.9%), upper airway abnormalities (UAAs) in 75 patients (21.7%), and chronic lower respiratory disease in 12 patients (3.5%) (Table 1).

**Table 1:** Demographic and clinical data

	*N* = 345
Characteristic	No. (%)
Sex	
Male	242 (70.1)
Female	103 (29.8)
Age (Median, IQR)	5 (3–8)
Background diseases:	
Global Developmental Delay	274 (79.4)
Autistic Spectrum Disorder	211 (61.1)
Seizure disorder	174 (50.4)
Upper Airway Abnormality	75 (21.7)
Genetic disorder	22 (6.3)
Lower respiratory disease	12 (3.5)
Heart defect	10 (2.9)

Concomitant medication use included antiepileptic drugs in 89 patients (25.8%), antipsychotic medications in 44 patients (12.7%), and stimulant medications for attention-deficit/hyperactivity disorder (ADHD) in 14 patients (4%).

A single bolus of DEX was sufficient to achieve adequate sedation in 276 patients (80%), while two boluses were required in 62 patients (18%), and three boluses in 7 patients (2%). A rescue dose of promethazine was administered in 8 patients (2.3%), of whom 4 (1%) were classified as sedation failures.

Notably, 18 patients with UAA (34% of this subgroup) required two or more doses of DEX, compared to 44 patients (15.1%) among those without UAA (P = 0.001).

The median total dose administered was 1.64 mcg/kg (IQR: 0.67 mcg/kg). The median time to achieve the target sedation level (sedation onset) was 5 min (IQR: 5 min), while the median time to complete recovery (sedation offset) was 90 min (IQR: 25 min). No significant differences in sedation onset or offset times were observed among patients with neurological, respiratory, cardiovascular, or genetic diseases.

The overall incidence of adverse effects was 8.4%. No sentinel adverse events were observed. The only moderate-severity adverse event documented was hypotension, which occurred in 6 patients (1.7%) and was successfully managed with intravenous fluid bolus administration, resulting in full recovery. Minor adverse effects included hypoxia in 6 patients (1.7%), stridor in 3 patients (0.9%), snoring in 4 patients (1.1%), and irritability in 10 patients (2.9%) (Table 2).

**Table 2:** Results and outcomes

	*N* = 345
Dexmedetomidine administration No. (%)	
No. of administered doses	
1	276 (80.0)
2	62 (18.0)
3	7 (2.0)
Total administered dose (mcg/kg) (Median, IQR)	1.64 (1.3–2)
**Sedation (Median)**	
Failure to achieve desired sedation level (MAAS-3) No. (%)	4 (1.1)
Time to goal MAAS (minutes) (Median, IQR)	5 (5–10)
Time to complete recovery (minutes) (Median, IQR)	90 (80–105)
**Adverse Effects No. (%)**	29 (8.4%)
SIVA severity- moderate:	
Hypotension	6 (1.7)
Bradycardia	0
Bradypnea	0
SIVA severity- minor:	
Hypoxia	6 (1.7)
Snoring	4 (1.1)
Stridor	3 (0.9)
SIVA severity- minimal:	
Irritability	10 (2.9)

MAAS-motor activity assessment scale

Patients with UAAs exhibited a trend toward a higher incidence of sedation-related adverse events compared to those without UAAs (13.2% vs. 6.5%, *P* = 0.095). Aside from this subgroup, the presence of other chronic illnesses was not associated with an increased risk of adverse events.

Importantly, a significantly higher incidence of adverse events was observed in association with increased cumulative doses of DEX. Patients who received more than one bolus had a substantially higher adverse event rate compared to those who received a single bolus (27.4% vs. 3.2%, *P* = 0.001). Similarly, when the total administered dose reached or exceeded 3 mcg/kg, the risk of adverse events increased markedly (40% vs. 6.1%, *P* = 0.001).

## Discussion

In this study, rapid bolus administration of DEX without continuous infusion appears to be a safe and effective sedation strategy for pediatric EEG procedures. This approach yielded a high sedation success rate (99%), with a rapid onset of sedation, predictable recovery times, and a low incidence of adverse events. Importantly, the protocol enabled efficient patient turnover, making it especially suitable for resource-limited clinical settings. Although DEX was well tolerated, individualized dose titration remains crucial to minimize the risk of adverse events, particularly in children with underlying UAA. These findings support the use of bolus-only DEX as a practical alternative to continuous infusion protocols, offering a simplified and time-efficient sedation strategy.

IV bolus administration of DEX has demonstrated high efficacy in achieving timely and reliable sedation in our study, with 80% requiring a single IV bolus and an extremely low failure rate (1%). Compared to other routes of administration, IV bolus had a markedly faster onset (5 min) and a predictable recovery time (90 min). In contrast, studies examining the clinical pharmacodynamics of IN DEX have reported longer onset times and higher failure rates. IN sedation typically requires 19–44 min for onset, with failure rates ranging from 15 to 17% [9–12]. A systematic review by Lewis et al. (2020), which included 14 randomized controlled trials on pediatric sedation with IN Dex, reported onset times of 15–30 min and durations of action ranging from 55 to 100 min [13]. Data on IM DEX are limited; however, Mason et al. published two retrospective studies evaluating its use for neuroimaging sedation. One study reported an average sedation onset of 13 min and a total sedation duration of 33–60 min until discharge criteria were met [8]. The second study reported an average sedation onset of 15.5 min (range: 3–55 min) and an average of 54.5 min to meet discharge criteria following EEG [14]. Notably, both studies achieved a 100% success rate in attaining adequate sedation. When examining traditional IV administration protocols involving a loading dose followed by continuous infusion, sedation onset is typically longer, ranging from 11.8 to 13.4 min, with total time from sedation onset to discharge ranging between 80 and 170 min [15, 16]. In this bolus-only protocol, the median total dose of DEX required to complete the procedure was 1.64 mcg/kg. This dosage is notably lower than previously reported doses, which range from 2.59 to 3.19 mcg/kg for comparable neuroimaging procedures [17, 18]. The reduced dosage may be attributed to the absence of continuous infusion, which might be unnecessary for procedures of medium duration.

In addition to its demonstrated efficacy, our IV bolus-only protocol was associated with a favorable safety profile, showing a notably low incidence of adverse events. The overall incidence of adverse effects was 8.4%, none of which were classified as sentinel events. The most significant adverse effects observed were hypotension (1.7%) and hypoxia (1.7%). These rates are considerably lower than those reported in studies employing traditional protocols involving an IV bolus followed by continuous infusion. For instance, Jooste et al. (2017) conducted a prospective study of 91 pediatric patients and reported 4.4% bradycardia, 41% hypotension, 2.2% hypoxia, and 2.2% vomiting [17]. Similarly, Khan et al. (2024) evaluated DEX monotherapy in an RCT of 122 pediatric patients undergoing MRI and reported 61% bradycardia, 24.6% hypotension, 8.2% hypoxia, and 2.4% vomiting [18]. The overall incidence of adverse events in those studies (74% and 92%, respectively) was substantially higher than in ours. However, it should be noted that both studies were prospective in design, with rigorous monitoring and documentation of adverse events, which may partly explain the discrepancy. Additionally, differing definitions of adverse events likely contribute to the variation in reported rates. In the studies by Jooste and Khan [17, 18], hypotension and bradycardia were defined as a 30% reduction from baseline, whereas in our study, hypotension was defined as a systolic blood pressure falling below the 5th percentile for age, and bradycardia as a heart rate below 60 bpm. In comparison to the safety profile of intramuscular DEX, Mason et al. reported an adverse event rate of 14% for hypotension, defined as a blood pressure decrease of more than 20% from the age-adjusted awake baseline value. Notably, no patients in that study experienced bradycardia, hypertension, or oxygen desaturation [8].

In the current study, patients with preexisting upper airway conditions (*n* = 53) including stridor, OSA, and tracheal anomalies, required higher doses of DEX to achieve adequate sedation. Only 35 of these patients (66%) achieved adequate sedation following a single bolus dose, compared to 85% of patients without upper airway pathology. Similarly, a previous study evaluating DEX as a sedative agent for drug-induced sleep cine-MRI in children with OSA reported even lower efficacy, with only 48% of participants achieving adequate sedation following a single bolus and continuous infusion [19]. These findings may reflect altered pharmacodynamic responses to DEX in this patient subgroup. Further research is warranted to determine whether alternative dosing strategies could enhance sedation success in children with upper airway abnormalities. This subgroup also exhibited a trend toward a higher incidence of sedation-related adverse events (P = 0.095). Although this trend did not reach statistical significance, it aligns with existing evidence indicating that pediatric patients with OSA have increased anesthesiological risk due to their heightened sensitivity to sedative and opioid medications, which can exacerbate airway obstruction and respiratory depression [20–22].

As expected, a significant correlation was observed between higher DEX doses and an increased risk of adverse events. These findings suggest that while bolus DEX is generally well tolerated, cautious dosing is warranted, particularly in patients with preexisting respiratory conditions.

Our findings have important clinical implications. The ability to achieve effective sedation with a single bolus dose simplifies sedation protocols, reduces drug exposure, and minimizes the need for prolonged monitoring associated with continuous infusions. Furthermore, the rapid onset and predictable recovery time enhance workflow efficiency in procedural and diagnostic settings.

## Limitations

This study has several limitations. First, it was a retrospective study without a control group, which limits the ability to establish causality or directly compare bolus administration with continuous infusion. Nonetheless, the comparison to published data on other dexmedetomidine protocols supports the current single-bolus approach. Second, it was conducted at a single center, which may have limited the generalizability of the findings. Additionally, study group is relatively small and no long-term outcomes were measured [23]. Future research should include larger, multicenter prospective trials with a control group to compare different dexmedetomidine dosing regimens and validate the safety and efficacy of bolus-only administration in pediatric sedation.

## Conclusions

To our knowledge, this is the first study to describe rapid bolus administration of dexmedetomidine without a continuous infusion for pediatric sedation. This strategy is effective and safe, providing rapid sedation onset, a predictable recovery profile, and a low incidence of adverse effects. Careful dose selection is advised to minimize the risk of adverse events, particularly in patients with upper airway abnormalities.

## Data Availability

The datasets generated and analyzed during the current study are not publicly available due to patient confidentiality but are available from the corresponding author on reasonable request.

## Abbreviations

ASD: Autistic spectrum disorder
CH: Chloral hydrate
DEX: Dexmedetomidine
EEG: Electroencephalography
GDD: Global developmental delay
IM: Intramuscular
IN: Intranasal
IV: Intravenous
IQR: Interquartile range
OSA: Obstructive sleep apnea
PEM: Pediatric emergency medicine
UAAs: Upper airway abnormalities
SIVA: World Society of Intravenous Anaesthesia
